# Current State of Pediatric Direct Primary Care: A National Cross-Sectional Survey of Pediatricians

**DOI:** 10.7759/cureus.84903

**Published:** 2025-05-27

**Authors:** Nicholas G Belt, Austin Lee, R Justin Mistovich, Andrew Hertz

**Affiliations:** 1 Medicine, Case Western Reserve University School of Medicine, Cleveland, USA; 2 Orthopedic Surgery, MetroHealth Medical Center, Cleveland, USA; 3 Pediatrics, Case Western Reserve University School of Medicine, Cleveland, USA

**Keywords:** direct primary care, financial viability, pediatricians, physician satisfaction, practice characteristics

## Abstract

Introduction

Direct primary care (DPC) is an emerging healthcare model emphasizing patient-centered care, reduced administrative burden, and enhanced physician satisfaction. While DPC has gained traction among pediatricians, limited data exist on the distribution, characteristics, and satisfaction of pediatric DPC practices. This study aims to provide a national overview of pediatric DPC practices, describe physician demographics and motivations, and evaluate satisfaction among DPC physicians.

Methods

We conducted a cross-sectional survey of US-based pediatricians practicing in the DPC model to assess physician demographics, practice characteristics, and satisfaction. A 19-section survey was developed in consultation with pediatric DPC leaders and piloted within the Zest Pediatric Network. It was distributed on July 11, 2023, via the “Pediatricians Who Do DPC” Facebook group, practice websites, and direct outreach. Physicians were eligible if ≥75% of their panel was pediatric and their practice was operational. Descriptive statistics were calculated, and satisfaction between current and prior roles was compared using a two-tailed t-test (p < 0.05). Analyses were performed using Microsoft Excel.

Results

The analysis included 73 pediatric DPC physicians across 26 states. Most practices were single-doctor models (85%), located in suburban settings (73%), and managed small patient panels (<200 patients: 79%). Physicians reported high satisfaction; 94% were happier in DPC than in prior roles, citing reduced moral injury (89%) and improved work-life balance. While 73% of physicians earned less in DPC initially, 65% with practices ≥3 years old reported incomes similar to or greater than prior positions. Physicians cited meaningful patient relationships, greater autonomy, and improved work-life balance as key benefits. Key challenges included financial uncertainty, marketing burdens, and isolation.

Conclusions

Pediatric DPC offers significant benefits, including high physician satisfaction and reduced administrative burden, despite initial financial challenges. As practices mature, financial viability improves, suggesting long-term sustainability. Future research should evaluate patient outcomes, financial accessibility, and strategies to support new practices.

## Introduction

Direct primary care (DPC) introduces a revitalized framework in healthcare, shifting from the traditional fee-for-service model toward a structure that prioritizes direct, enduring relationships between physicians and patients [1]. Unlike conventional care models, where high patient volumes, intricate billing processes, and administrative demands can impede accessible and efficient care, the DPC approach sidesteps these barriers [2,3]. DPC operates on a membership-based subscription model, where patients pay a regular monthly fee directly to the physician’s practice, eliminating the need for co-pays and insurance-related expenses [4]. This model enables physicians to focus more extensively on providing patient-centered, evidence-based care rather than billing and administrative tasks. DPC practices often incorporate modern telehealth services alongside in-office visits, facilitating broader access and enhancing patient satisfaction. The model’s emphasis on building lasting, longitudinal professional relationships with patients seeks to improve healthcare quality, reduce overall costs, and deliver higher standards of care.

In recent years, there has been growing interest in DPC among pediatricians who are drawn to its patient-centered approach and the potential for a more sustainable, fulfilling practice environment [5,6]. Despite this rising trend, data on the distribution, structure, and practice-specific value propositions of pediatric DPC practices remain limited. Similarly, there is little understanding of the background and motivations of pediatricians who choose to transition to this model, nor is there ample information regarding their satisfaction within the DPC model compared to their prior satisfaction in a traditional practice. Specifically, the objectives of this study were to (1) characterize the current landscape and operational attributes of pediatric DPC practices across the United States, (2) examine the demographics and professional profiles of physicians leading these practices, and (3) evaluate physician satisfaction levels within the DPC model relative to prior experience in conventional pediatric practice. By examining these elements, this study seeks to provide a comprehensive, national baseline perspective on the current state of pediatric DPC.

## Materials and methods

Study design

This study utilized a cross-sectional survey design to assess physician demographics, practice characteristics, and physician satisfaction among pediatricians practicing within the DPC model. The survey was developed and administered using Google Forms (Alphabet Inc., Mountain View, CA).

Survey development and data collection

A comprehensive 19-section survey was developed internally by our research team after semiformal discussions with pediatricians actively involved in the DPC community. Initial input on survey content and relevant variables was obtained through discussions with pediatric DPC doctors, particularly leveraging the expertise of the pediatric DPC mastermind leadership team. Additionally, preliminary survey content was presented at a lecture during a pediatric DPC mastermind meeting, allowing attendees to suggest improvements before final deployment. 

Prior to formal dissemination, the survey underwent beta pilot testing within the Zest Pediatric Network. Feedback was solicited specifically on content relevance, clarity of questions, and completion time. This pilot phase informed refinements to enhance survey understandability and practicality.

Several steps were taken to enhance survey validity and reliability. Each respondent was permitted only a single survey submission, enforced electronically. Clear inclusion criteria restricted participation exclusively to pediatricians actively practicing in a pediatric-focused DPC model, ensuring homogeneity and relevance of responses. Additionally, survey design was informed by existing literature on adult-focused DPC practices, with intentional adaptations to address pediatric-specific nuances not previously explored. 

The survey was disseminated on July 11, 2023, through multiple channels. It was shared in the “Pediatricians Who Do DPC” Facebook group, which serves as an online community for DPC pediatricians. Additionally, direct email invitations were sent to pediatric DPC practices using publicly available email addresses obtained from practice websites. Further outreach was conducted by emailing pediatric DPC physicians whose contact information had been obtained by the authors through professional networking. The survey was sent to approximately 130 physicians, and it remained open for three weeks to allow sufficient time for responses.

Inclusion and exclusion criteria

Physicians practicing in a pediatric DPC setting within the United States were eligible to participate. Responses were excluded if the respondent was not a physician, if their practice was not yet operational at the time of the survey, or if they provided care to both pediatric and adult patients and more than 25% of their patient panel consisted of adults (≥18 years of age).

Statistical analysis

Descriptive statistics were used to summarize physician and practice characteristics. A two-tailed t-test was performed to compare physician satisfaction between their previous job and their current role in DPC. A p-value of <0.05 was considered statistically significant. All statistical analyses were conducted using MS Excel (Microsoft Corporation, Redmond, Washington, United States).

## Results

Current state of DPC practices

**Figure 1:**
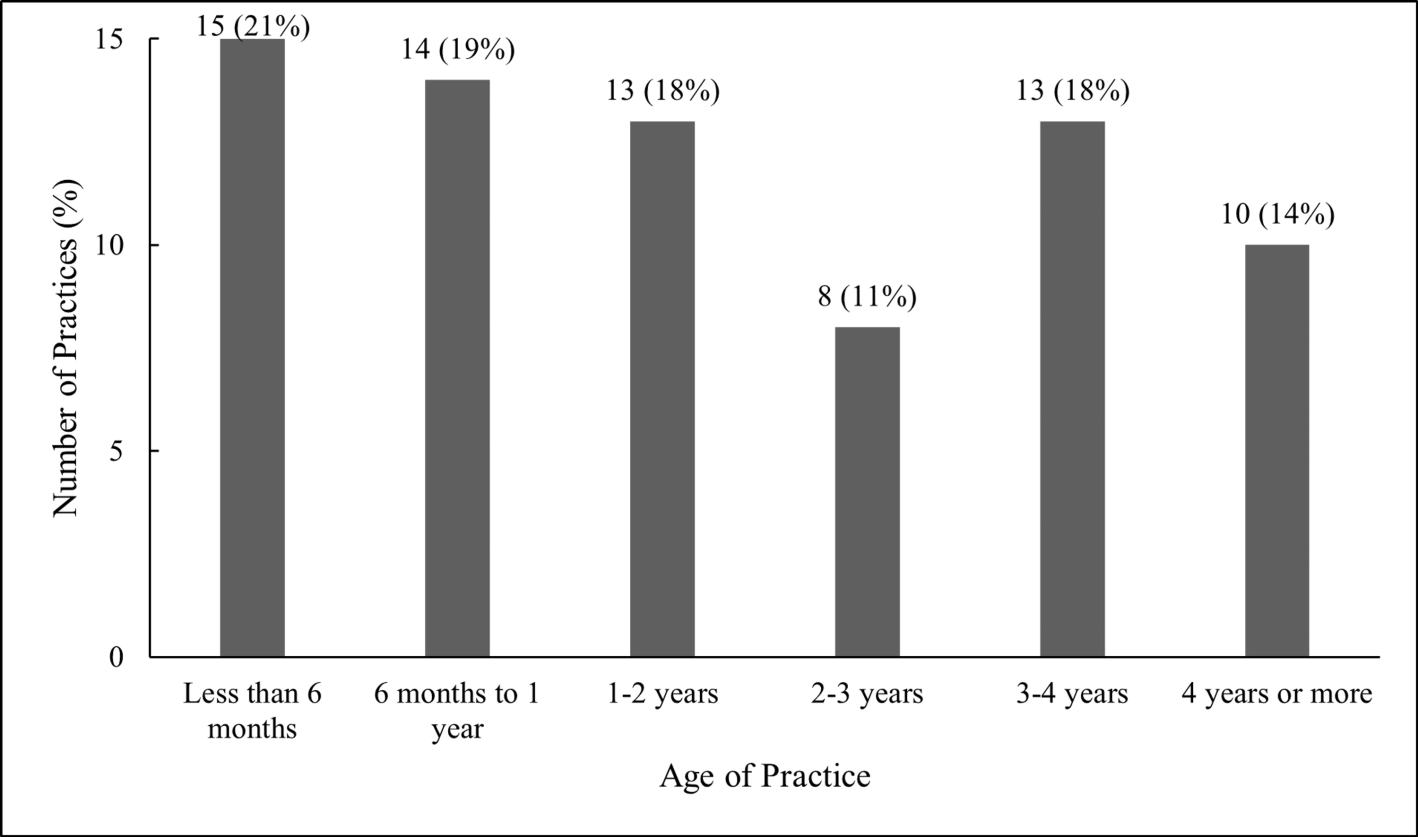
Age of practices

Initial responses were obtained from 79 pediatricians out of the 130 surveyed, yielding a 61% response rate. Six responses were excluded because the practices were either not yet operational or saw less than 75% pediatric patients. This resulted in a final analytic cohort of 73 pediatricians with unique pediatric DPC practices, corresponding to a 56% effective response rate. These practices are in 26 different states, with Florida (13 practices), Texas (11 practices), and Ohio (six practices) being the most represented. Most of these practices are located in suburban areas (53, 73%), followed by rural (12, 17%) and urban (7, 10%) settings. The maturity of these practices varies, with a notable proportion being relatively young (Figure 1); 29 (40%) have been operational for less than one year, while only 10 (14%) have been operational for four years or more.

**Figure 2:**
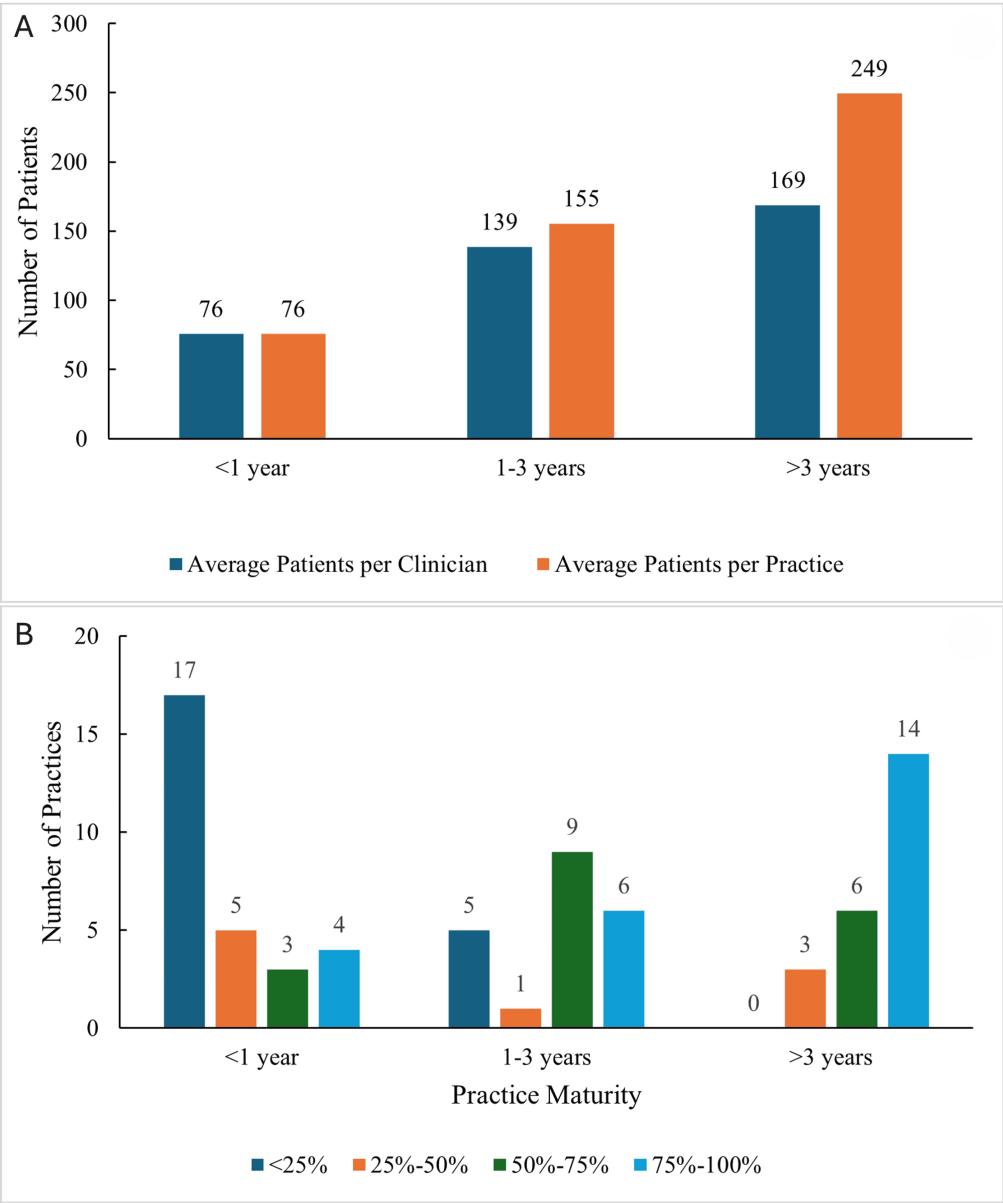
Number of patients per physician and current achievement of patient goal per physician based on practice maturity (A) Average number of patients per clinician and per practice, stratified by practice maturity (<1 year, 1–3 years, and >3 years). (B) Distribution of practices by the percentage of panel size goal achieved, stratified by practice maturity. Panel size categories include <25%, 25%-50%, 50%-75%, and 75%-100%

Most of the practices are relatively small, with 62 (85%) of them being single-doctor practices (Table 1) and 34 (47%) having no additional staff (Table 2). A total of 39 (53%) of the practices that employ at least one staff member typically have a medical assistant or office manager. Most patient panels are modest in size (Table 1). At the time of the survey, 33 (45%) of the physicians manage less than 100 patients, 25 (34%) manage 100 to 200 patients, 11 (15%) manage 200 to 300 patients, and only four (5%) manage more than 300 patients. Additionally, most physicians aim for smaller patient cohorts, with 19 (26%) reporting a goal of less than 150 patients per physicians, 29 (40%) reporting a goal of 151 to 250 patients, 16 (22%) reporting a goal of 251 to 350 patients, and nine (12%) aiming for greater than 350 patients. As practices mature, pediatricians tend to manage larger patient panels (Figure 2). Young practices (open less than one year) have a mean census of 76 patients per physician, maturing practices (open 1-3 years) have an average of 139 patients per physician, and mature practices (open more than three years) have an average of 169 patients per physician. Practices achieve a higher proportion of their patient panel goals as they age (Figure 2).

**Table 1:** DPC practice characteristics DPC: direct pediatric care

Practice characteristics		n (%) (N = 73)
Area of practice		
	Urban	7 (10)
	Suburban	53 (73)
	Rural	13 (17)
Age of practice		
	Less than 6 months	15 (21)
	6 months to 1 year	14 (19)
	1-2 years	13 (18)
	2-3 years	8 (11)
	3-4 years	13 (18)
	4 years or more	10 (14)
Number of doctors in the practice		
	1	62 (85)
	2	9 (12)
	3	2 (3)
Number of current patients per physician		
	<100	33 (45)
	100-200	25 (34)
	200-300	11 (15)
	>300	4 (5)
Goal for patients per physician		
	150 or less	19 (26)
	151-250	29 (40)
	251-350	16 (22)
	Greater than 350	9 (12)
Type of enrollment fee		
	Fee per family	44 (60)
	Fee per patient	11 (15)
	None	18 (25)
Average monthly membership per patient		
		8 (11)
	$101-125	21 (29)
	$126-150	21 (29)
	$150-175	15 (21)
	>$175	7 (10)
Medicaid patients		
	Yes	51 (70)
	No	22 (30)
Service delivery		
	Only home visits	7 (10)
	Only office visits	6 (8)
	Combination	60 (82)
Vaccination delivery		
	Vaxcare	47 (64)
	Obtain from a local pharmacy and delivery myself	17 (23)
	Vaccine for children	18 (25)
	Purchase vaccines and cover the cost	8 (11)
	Purchase vaccines and pass the cost onto the patient	3 (4)
	Send patient to another practice or obtain vaccine from another practice	7 (10)
	I do not provide vaccines	3 (4)
	Health department	8 (11)
	Other vaccine vendor	3 (4)
Do you have a cash pay contract for an outside lab?		
	Yes	39 (53)
	No	34 (47)
Do you have a cash pay contract for outside imaging?		
	Yes	7 (10)
	No	66 (90)
Regular days or hours that the office is closed		
	Yes	25 (34)
	No	48 (66)
When on vacation and a patient needs to be seen in-person, who provides coverage		
	Other providers in the practice	15 (21)
	Another pediatric DPC doctor	32 (44)
	Another DPC doctor	12 (16)
	Locum	8 (11)
	No one, I tell families to use urgent care or emergency department if clinically indicated	23 (32)

**Table 2:** Employment of support staff among DPC practices DPC: direct pediatric care; MA: medical assistant; LPN: licensed practical nurse; RN: registered nurse; NP: nurse practitioner; PA: physician assistant

Staff	Employed	None employed
	n (%) (N = 73)	n (%) (N = 73)
Virtual assistant	7 (10)	66 (90)
Secretary/receptionist	10 (14)	63 (86)
MA	12 (16)	61 (84)
LPN	3 (4)	70 (96)
RN	7 (10)	66 (90)
NP or PA	8 (11)	65 (89)
Office manager	13 (18)	60 (82)
Any of the above	39 (53)	34 (47)

When assessing the cost for patients and families, most (44, 60%) practices implement a family-based enrollment fee, rather than a per patient (11, 15%) or no enrollment fee (18, 25%). The range of monthly membership costs spans from $25 to $400, with most practices charging between $101 and $150; eight (11%) practices charge less than $100, 21 (29%) practices charge $101-$125, another 21 (29%) charge $126-$150, 21% charge $151-$175, and seven (10%) practices charge more than $175. The outliers on both ends of the spectrum have distinct characteristics. The lowest-cost practice ($25 per month) is in Michigan, is less than a year old, and has relatively fewer patients. On the higher end, practices charging $250 and $400 are in higher-cost-of-living states (California and Florida), are more mature (operating for over four years), and have modest patient panels. Seventy percent of practices reported providing care to Medicaid-insured children. Most practices (67, 92%) reported providing home-based care (Table 1), with seven (10%) providing exclusively in-home care.

The range of labs and services offered varied greatly between the practices, and a thorough overview can be found in Table 3. The most common laboratory tests included with a membership were urinalysis (69, 95%), rapid strep (69, 95%), serum glucose (55, 75%), and pregnancy (53, 73%). The most common services included with a membership were hearing screening (53, 73%), laceration gluing of any type (54, 74%), suturing of any type (41, 56%), and splinting of any type (29, 40%). Seventy (96%) practices also provide vaccines (Table 1), with the third-party vendor Vaxcare (Orlando, FL) being the most common solution for vaccine provision (47, 64%).

**Table 3:** Labs and services provided U/A: urinalysis; RSV: respiratory syncytial virus; CBC: complete blood count

		Included in membership	Extra charge	Do not offer
Labs		n (%) (N = 73)	n (%) (N = 73)	n (%) (N = 73)
	U/A	69 (95)	2 (3)	2 (3)
	Pregnancy	53 (73)	1 (1)	19 (26)
	Rapid strep	69 (95)	4 (5)	0 (0)
	Rapid flu	56 (77)	7 (10)	10 (14)
	Rapid RSV	35 (48)	9 (12)	29 (40)
	Rapid COVID	51 (70)	7 (10)	15 (21)
	Rapid respiratory panel	6 (8)	14 (19)	53 (73)
	Other rapid antigen panel	6 (8)	8 (11)	59 (81)
	Lead	17 (23)	9 (12)	47 (64)
	Hemoglobin	44 (60)	6 (8)	23 (32)
	Cholesterol	7 (10)	12 (16)	54 (74)
	CBC	4 (5)	12 (16)	57 (78)
	Mono	18 (25)	10 (14)	45 (62)
	Transcutaneous bilirubin	7 (10)	5 (7)	61 (84)
	Serum glucose	55 (75)	2 (3)	14 (19)
	Occult hemoglobin	46 (63)	3 (4)	22 (30)
Services		n (%)	n (%)	n (%)
	Electronic vision screening	22 (30)	0 (0)	51 (70)
	Hearing screening	53 (73)	0 (0)	20 (27)
	Suturing of any type	41 (56)	9 (12)	23 (32)
	Gluing of any type	54 (74)	8 (11)	11 (15)
	Splinting of any type	29 (40)	6 (8)	38 (52)
	Casting of any type	4 (5)	2 (3)	67 (92)
	Ear piercing	4 (5)	46 (63)	23 (32)
	Medication dispensing	10 (14)	20 (27)	43 (59)
Additional Services		n (%)	n (%)	n (%)
	Autism spectrum disorder testing	20 (27)	-	53 (73)
	One-time consults	40 (55)	-	33 (45)
	One-time Visits	33 (45)	-	40 (55)
	Behavior health only plans (ADHD, anxiety, depression, etc.)	22 (30)	-	51 (70)
	Fourth trimester package	24 (33)	-	76 (67)
	Lactation consultation only	13 (18)	-	60 (82)
	Community vaccine clinic	29 (40)		44 (60)

Characteristics of physicians in DPC practices

The surveyed physicians predominantly have MD degrees (63, 86%) and are board certified by the American Board of Pediatrics (62, 85%) (Table 4). The gender distribution of physicians in these practices is notably skewed toward females (60, 82%). The age distribution shows that most physicians are in the 40-49 age range (41, 56%), followed by 30-39 (18, 25%) and only three (4%) aged 60 or above.

**Table 4:** Physician characteristics

Physician characteristics		n (%) (N = 73)
Training		
	MD	63 (86)
	DO	10 (14)
Gender		
	Male	13 (18)
	Female	60 (82)
Age		
	Under 30	0 (0)
	30-39	18 (25)
	40-49	41 (56)
	50-59	11 (15)
	60 and over	3 (4)
Board certification		
	American Board of Pediatrics	62 (85)
	American Board of Osteopathic Pediatrics	3 (4)
	National Board of Physicians and Surgeons	1 (1)
	Not board certified	7 (10)
Prior job		
	Employed by a hospital or health system	32 (44)
	Employed by a private group	18 (25)
	Partner in a private group	15 (21)
	Owner of solo private practice	5 (7)
	Non-general peds employed by a hospital	3 (4)
Prior job hours		
	Full-time	47 (64)
	Part-time	26 (36)

Most physicians were previously employed by either a hospital or health system (32, 44%) or in a private group (18, 25%). Some were partners in private groups (15, 21%). The rest of the respondents were either in solo fee-for-service practices (5, 7%) or were not providing primary care (3, 4%). The prior jobs also tended to be full-time positions (47, 64%).

Physician satisfaction

**Figure 3:**
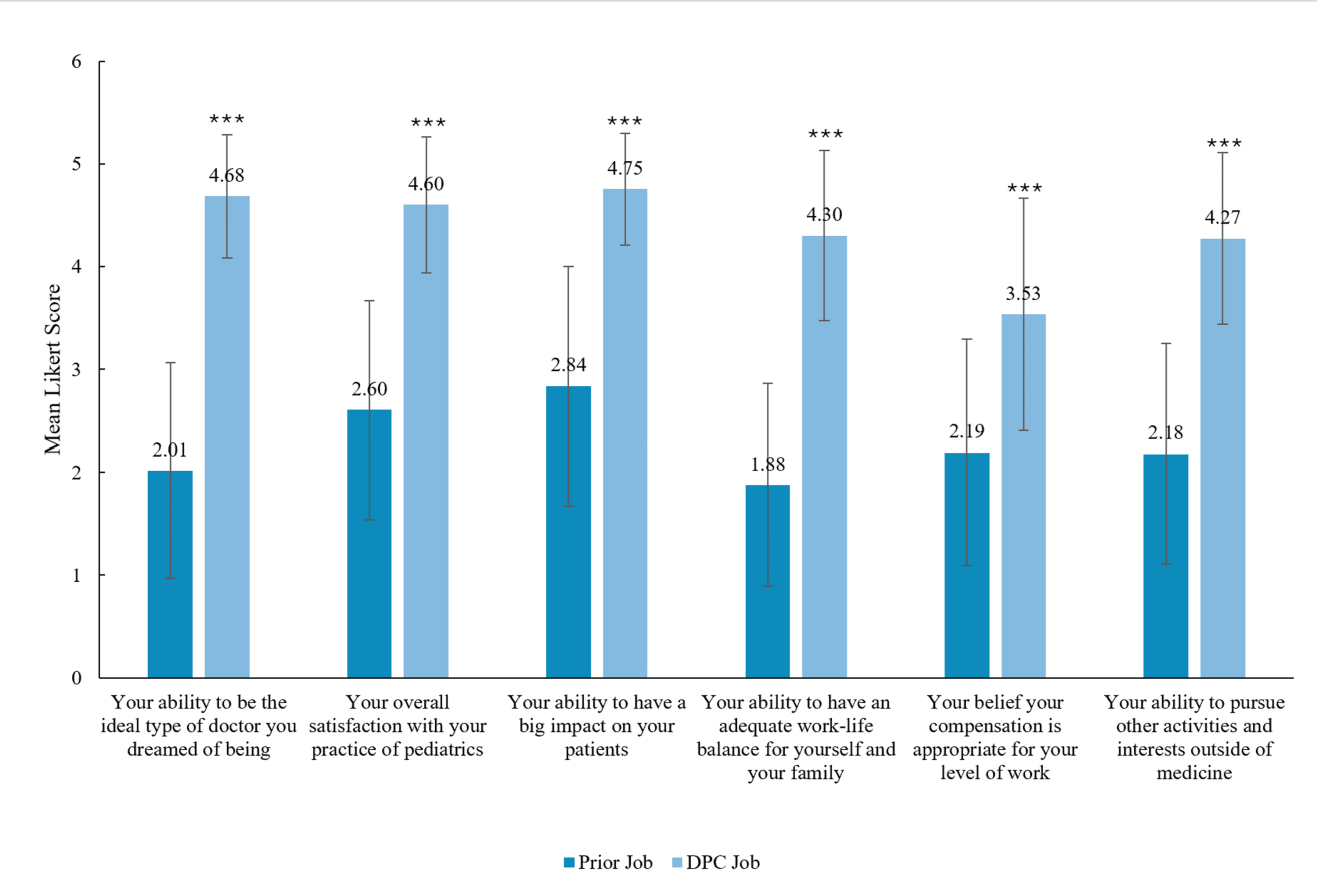
Comparison of satisfaction between prior and current job based on mean five-point Likert scale DPC: direct pediatric care Mean satisfaction scores are shown for six domains comparing respondents' prior pediatric jobs with their current DPC jobs. Satisfaction was rated on a five-point Likert scale, with higher scores indicating greater satisfaction. Error bars represent ±1 standard deviation. Statistical comparisons were conducted using paired two-tailed t-tests. Asterisks (***) indicate statistical significance with ***p < 0.001

Professional satisfaction in DPC practices is high, especially when compared to the respondents’ previous jobs (Table 5). Nearly all physicians (69, 94%), reported being happier in their DPC practice than in their prior job, with 39 (53%) physicians stating they are “far more happy.” We found a statistically significantly higher level of job satisfaction in their current DPC practice compared to their prior job across various metrics, including their ability to practice the ideal type of medicine they envisioned, overall job satisfaction, and work-life balance (Figure 3). Additionally, 65 (89%) reported experiencing less or far less moral injury compared to their previous job.

**Table 5:** Physician satisfaction DPC: direct pediatric care

Physician satisfaction		n (%) (N = 73)
Weeks of vacation per year (only practices open for >1 year) (N = 46)		
	1 week	3 (7)
	2 weeks	5 (11)
	3 weeks	16 (35)
	4 weeks	17 (40)
	5 weeks	1 (2)
	6 weeks	3 (7)
	9 or more weeks	1 (2)
How would you describe that amount of vacation? (N = 46)		
	Too little	19 (41)
	About right	27 (59)
	Too much	0 (0)
How does your DPC practice income compare to your previous job?		
	Less	53 (73)
	About the same (within $10,000)	11 (15)
	More	9 (12)
Describe your overall level of happiness compared to your previous job?		
	Less happy	1 (1)
	About the same	3 (4)
	More happy	30 (41)
	Far more happy	39 (53)
Rate your current level of moral injury compared to your previous job		
	Far Less Moral injury now	51 (70)
	Less moral injury now	14 (19)
	About the same moral injury now	8 (11)
	More moral injury now	0 (0)
	Far more moral injury now	0 (0)
Would you ever consider returning to fee-for-service primary care pediatrics?		
	Yes	2 (3)
	Maybe	15 (21)
	No	56 (77)

**Figure 4:**
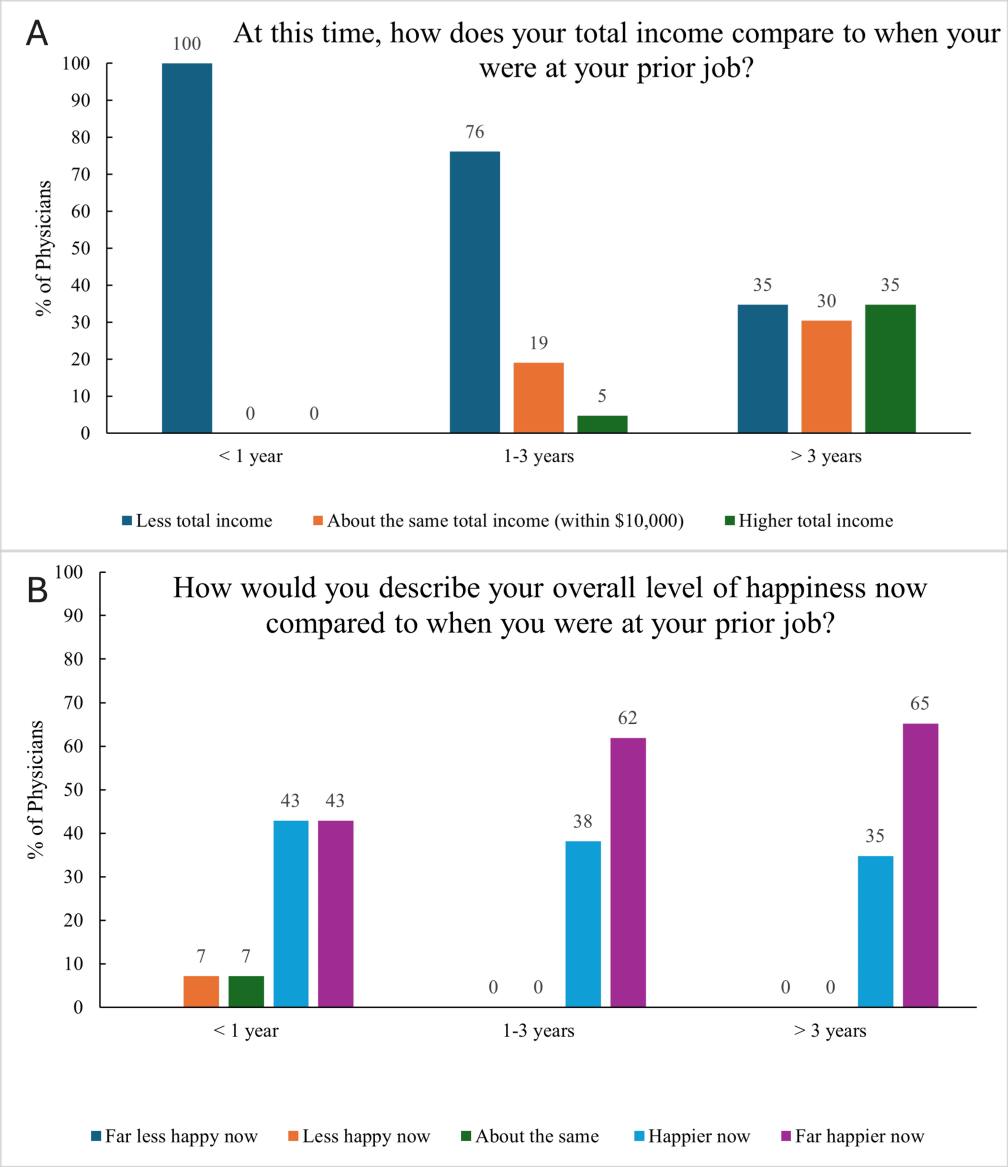
Comparison of income and happiness between prior and current job based on practice maturity DPC: direct pediatric care (A) Percentage of physicians reporting whether their current total income is lower, about the same (within $10,000), or higher compared to their income at their prior job, grouped by how long their DPC practice has been open (<1 year, 1–3 years, >3 years). (B) Percentage of physicians describing their current overall happiness compared to their prior job, using five categories ranging from “Far less happy now” to “Far happier now,” also grouped by practice maturity. Panels A and B are labeled in the upper left corner of each panel for clarity and reference

Notably, 53 (73%) physicians indicate that their income is lower in their DPC practice compared to their previous job. However, for those physicians working in practices open three or more years, 47 (65%) reported having similar or greater total income than in their prior job (Figure 4).

Only two (3%) physicians responded that they would consider returning to a fee-for-service primary care position. Among practices that have been open for more than a year, 29 (40%) physicians reported taking four weeks of vacation annually. Most respondents believe that their current amount of vacation is appropriate (43, 59%).

When asked in a free response format about positive aspects of DPC, common themes included finding meaning in developing personal relationships with patients and families, better flexibility and autonomy, enhanced quality of care, and improved work-life balance. Conversely, when asked about the challenges, common themes included uncertainty in building and maintaining their practice, administrative burdens related to marketing and operational tasks, managing patient expectations and boundaries, uncertainty of the practice’s viability, and isolation due to being a sole practitioner.

## Discussion

Our study offers the first comprehensive overview of the current state of pediatric DPC pediatricians and their practices in the US. The number of pediatric DPC practices has increased across the country in recent years. Our results align with previous studies, which found that most DPC practices are relatively new [2,3]. This growth is driven by DPC’s unique benefits, such as fostering stronger physician-patient relationships, reducing administrative burden, and increasing physician satisfaction [4,5]. In line with previous literature, we found that most DPC practices operate with small staff sizes and usually have a single physician [7]. Our results also reveal the challenges related to building a business and ensuring affordability and accessibility for all patients.

One of the most significant findings in this study is the high level of physician satisfaction within pediatric DPC practices. Nearly all respondents (69, 94%) reported feeling more satisfied in their DPC roles compared to their previous roles. This finding aligns with Page et al., who found that 82% of physicians in direct pay models report increased satisfaction [8]. Additionally, previous literature outlined that the reduced administrative workload, greater autonomy, and improved work-life balance were seen as major advantages to the DPC model [7,9].

The financial structure of DPC practices typically involves a flat monthly fee that covers all of the patient’s primary care with no additional out-of-pocket charges [5]. This appeals to both physicians and patients as it simplifies billing and increases cost transparency. However, the affordability and accessibility of DPC memberships remain a point of concern, especially for low-income patients [3]. Notably, 51 (70%) practices in our study reported caring for Medicaid patients. This suggests that some DPC clinics may be accessible to diverse socioeconomic groups. However, since the financial demographics of DPC patients were not comprehensively analyzed in this study, further investigation is needed to evaluate whether these practices are truly accessible to lower-income families.

DPC’s ability to enhance physician well-being is particularly important given the increasing concerns about burnout in medicine. Ortega et al. found that physician burnout rates have increased from 44.4% in 2017 to 50.4% in 2021 [10]. Studies have shown that these increasing rates are being driven by loss of autonomy [11], high workload [12], increased bureaucratic tasks [13], and poor work-life balance [14]. By reducing or eliminating these burdens, DPC practices not only allow physicians to enjoy their work but also improve the quality of care provided to patients [12,14]. This study further indicates far lower burnout (moral injury) felt by DPC pediatricians. Our data demonstrated that 60 (82%) of the pediatricians were women, more than the national average of 72% [15]. This difference may be considered an indication and result of the increased challenges facing women in a conventional practice model.

Despite the high rates of professional satisfaction with the DPC model, financial challenges remain a concern for many physicians. A total of 53 (73%) physicians reported earning less in their DPC practice compared to their previous employment, though this number is likely skewed by the large proportion of young practices among the respondents. In fact, 47 (65%) physicians in DPC for three or more years reported the same or greater income. Interestingly, despite these financial metrics, only two (3%) physicians expressed they would consider returning to a fee-for-service model in the future. This highlights the significance of the nonmonetary benefits that the DPC model provides to physicians.

An additional challenge noted in the survey was that DPC pediatricians may feel isolated, especially in solo practices, which can lack the support found in larger healthcare organizations. To address these limitations, DPC networks and professional organizations can play a role in providing peer support, resources, and shared best practices to help promote long-term success.

Our survey outlines some major differences between DPC for pediatricians from the characteristics of general DPC practices described in the literature [9,16]. In general, these practices have smaller panel sizes, have higher membership prices, and perform home visits (Table 6). These differences, along with many potential other metrics such as the number of times parents access the pediatrician for their children compared to their own physician, may ultimately demonstrate that pediatric DPC practices are substantially different from adult-focused DPC practices. These differences have implications for workforce development, practice finances, physician education and support, and future studies. As such, we suggest distinguishing these differences by calling pediatric-specific DPC offices as Direct Pediatric Care. 

**Table 6:** Adults vs pediatric focused practices Table credits: Dr. Andrew Hertz and Nicholas Belt

	Direct pediatric care	Direct primary care
Percentage of patients less than 18 years old	100%	18%
Panel size	100-300	300-600
Average membership for children per month	$100-$175	$20-$75
Home visits	Yes	Maybe

Limitations

While this study provides valuable insights into the current state of pediatric DPC practices, there are several limitations. The total number of pediatric DPC practices in the country is not known, so it is unclear what percentage of total practices the 73 respondents represent. As a result, these findings may not be generalizable to all pediatric DPC practices. However, based on the best available data, we believe the total number of pediatric practices in the US was slightly over 100 at the time of the survey, indicating a rather high response rate of nearly 70%. Additionally, the survey was promoted through specific online platforms and email lists, which may have introduced a selection bias, as physicians who are more engaged in DPC-related communities may be more likely to participate in the study, though we suspect this limitation may be similar for all survey-related study designs. Second, the survey relied on self-reported data, which introduces the potential for response bias. Physician responses regarding their satisfaction, income, or patient panel size may have been impacted by social desirability bias or recall bias. Third, the financial demographics of patient populations were not comprehensively analyzed in this study. Future research should investigate the composition of patient financial groups within pediatric DPC practices to determine whether these practices are effectively serving patients across a diverse range of socioeconomic backgrounds. Despite these limitations, this study contributes valuable information to the limited body of literature on DPC, particularly in the pediatric population, and highlights the potential benefits and challenges that this model poses to pediatricians.

## Conclusions

This national survey provides the first comprehensive snapshot of pediatricians practicing in the DPC model across the US. The data highlight that pediatric DPC practices are typically small, often solo-operated, and commonly located in suburban or rural settings. Despite early financial challenges, physicians overwhelmingly report high professional satisfaction, reduced moral injury, and improved work-life balance within this model. Notably, satisfaction tends to increase as practices mature, with many reporting income improvement over time. The findings underscore the appeal of DPC among pediatricians seeking greater autonomy and deeper patient relationships. While challenges such as marketing, financial uncertainty, and professional isolation persist, the model appears to offer a sustainable and fulfilling alternative to traditional pediatric practice. Future research should explore long-term patient health outcomes, practice financial viability, and strategies to enhance accessibility for diverse patient populations.

## References

[1] Cole ES (2018). Direct primary care: applying theory to potential changes in delivery and outcomes. J Am Board Fam Med.

[2] Tou LC, Jeyakumar SJ, Siddiqui TA, Ravi S, Prakash N (2022). Understanding patient perceptions towards direct primary care: a focus group study. J Patient Exp.

[3] Eskew PM, Klink K (2015). Direct primary care: practice distribution and cost across the nation. J Am Board Fam Med.

[4] Kauffman RD (2020). Transitioning to direct primary care. Fam Pract Manag.

[5] Huff C (2015). Direct primary care: concierge care for the masses. Health Aff (Millwood).

[6] M. D. Andrew R. Hertz (2024). Direct primary care a growing model for pediatricians. https://publications.aap.org/aapnews/news/21981/Direct-primary-care-a-growing-model-for.

[7] Brekke G, Onge JS, Kimminau K, Ellis S (2021). Direct primary care: family physician perceptions of a growing model. Popul Med.

[8] Page L (2013). The rise and further rise of concierge medicine. BMJ.

[9] (2024). The pros and cons of direct primary care (DPC). https://www.wolterskluwer.com/en/expert-insights/what-exactly-is-direct-primary-care.

[10] Ortega MV, Hidrue MK, Lehrhoff SR (2023). Patterns in physician burnout in a stable-linked cohort. JAMA Netw Open.

[11] Fred HL, Scheid MS (2018). Physician burnout: causes, consequences, and (?) cures. Tex Heart Inst J.

[12] Shanafelt TD, Dyrbye LN, Sinsky C, Hasan O, Satele D, Sloan J, West CP (2016). Relationship between clerical burden and characteristics of the electronic environment with physician burnout and professional satisfaction. Mayo Clin Proc.

[13] Ariely D, Lanier WL (2015). Disturbing trends in physician burnout and satisfaction with work-life balance: dealing with malady among the nation's healers. Mayo Clin Proc.

[14] Shanafelt TD, Balch CM, Bechamps G (2010). Burnout and medical errors among American surgeons. Ann Surg.

[15] (2024). Pediatricians’ practice and personal characteristics. https://www.aap.org/en/research/periodic-survey-of-us-aap-members/pediatricians-practice-and-personal-characteristics/.

[16] (2024). Direct primary care. https://www.aafp.org/family-physician/practice-and-career/delivery-payment-models/direct-primary-care.html.

